# Causes of Death in Strangulated Hernia1Read at the thirty-fourth annual meeting of the Medical Association of Central New York, held at Buffalo, N. Y., August 27 and 28, 1901.

**Published:** 1901-12

**Authors:** William B. Jones

**Affiliations:** Rochester, N. Y.


### Causes of Death in Strangulated Hernia.[1]

By WILLIAM B. JONES, M. D., Rochester, N. Y.

"HE HAD a hernia for many years, it came out and he died. It had been down a good many times before, but he had always got it back;" that is the way it is usually told, but it conveys no idea of how it killed him, and I think many physicians have no definite conception of how strangulated hernia does kill. Without intelligent comprehension of what may happen, neglect, procrastination or misdirected effort usually sacrifices a life.

Reflection upon the fatal conditions resulting from strangulation surprises one at their number. Nearly always there is shock, often seveie and occasionally fatal. It is liable to be extreme in elderly people and may be aggravated by taxis, or by carrying the patient a distance, if the accident occurs where that is necessary. It increases too with the length of time before

1. Read at the thirty-fourth annual meeting of the Medical Association of Central New York, held at Buffalo, N. Y., August 27 and 28, 1901.

relief is obtained. It increases the danger of operation and should be prevented. How to do so suggests itself, but to do it should be in mind from the beginning. Rare exceptions are found and every once in a while a case turns up, having had what was mistaken for acute indigestion, a bilious attack or obstinate constipation but without the usual amount of shock, and after three, four or seven days, a hernia is found that had been there all the time not only strangulated, but also gangrenous and ruptured, leaking feces. Shock is also greatly increased by taxis.

The strangulated loop becomes inflamed when the constriction only embarrasses the circulation, but when the blood supply is stopped gangrene occurs at once. In either case it rapidly becomes septic and infects the surrounding tissues. From there by extension, general peritonitis follows, which is septic and rapidly fatal. Nature's method to prevent that is to confine this inflammation by adhesions about the ring until suppuration works an opening through the skin, allowing the formation of an artificial anus.

There may be leakage from torn intestine directly into the abdomen. The tearing of the gangrenous portion may be on the proximal side of the ring where feces escape into the abdomen, or having bursted outside the ring, it may be withdrawn into that cavity by the traction of the muscular fibers of the mesentery and bowel. This traction is always noticed during operations, to draw back the bowel as soon as it is liberated by section of the constriction, sometimes moving it several inches, and that even after the exhaustion of being a few days in that condition. It is greatest when the accident has just occurred and is the cause of the agonising, sickening pain and shock. It has sometimes torn the rest of the bowel away from the hernia, and that is another way leakage of feces has occurred. It is another of nature's resources, because it spontaneously reduces some hernia and has been known to do so after taxis has failed, and it assists in all reductions that do not come to operation.

The hernia must be freed from constriction, either by passing it back through the canal and ring, or by cutting the latter, and it must also be viable when reduced. If it be reduced, whether by taxis or by operation, there are several unfortunate possibilities. No one operates at the present without opening the sac to ensure relief at the ring, but taxis does replace some of them, sac and all, unrelieved. After incision it is often difficult to judge of the viability of the injured part and when taxis is successful we know nothing about it. An error of judgment in the first case or misfortune in the second, and subsequent sloughing finishes a short case history.

Functional obstipation causes death. In a very small hernia consisting of a part from a side of the bowel, there is not a real occlusion, but the effect is the same. There is no vermicular contraction opposite the portion that is pinched and no movement of feces past that point. The usual symptoms of intestinal obstruction lead to the usual termination of that condition. Also when hernia of the whole circumference of the bowel has caused paralysis, localised in that part without sloughing and that is returned into the abdomen, the paralysis remaining, the patient dies of inaction of the bowels. I know of no way to tell this condition in doubtful cases, the tissues are alive, but the distention does not disappear, and many deaths can only be explained in this way. It should be remembered that paralysis in a portion of the intestine means vaso-motor paralysis as well; the congestion means less ability to resist the erosion of fecal matter and the penetration of microörganisms, besides the epithelium is not readily renewed after such injury and the progress is, invasion of mucous membrane by bacteria, desquamation in patches, leaving ulcers through which germs and toxins readily enter, but are absorbed into the circulation and partly pass through the paretic congested bowel into the peritoneal cavity, causing an especially rapid fatal septicemia and peritonitis. Nor is this embarrassment of circulation and other function limited to the hernia. If we look for it we often find a number of inches of intestine a distance of some inches from the hernia paretic and distended and in this portion the mucous membrane would show a large area of deepest congestion with many sloughing ulcers. Fatal peritonitis results from infection entering these areas and passing right through the bowel wall into the peritoneum. Hemorrhage from the same place has taken off many who would have recovered but for that. The condition is due to traction and twisting of the mesentery, choking the blood vessels.

Mrs. B., aged 63, was well until six days before I saw her. At that time while coughing she suddenly had a severe pain in the left groin and found a small swelling, which soon became smaller, but had not disappeared. After about two days of severe colicky pain with vomiting, the former ceased and the only symptoms were vomiting and inability to move the bowels.

Loss of strength was very gradual and at my first visit her condition was surprisingly good. Operation within two hours showed one place where the small intestine had been constricted and still had a narrow ring of ecchymosis, but it had been withdrawn early and had preserved its physiological as well as anatomical integrity. The portion found unreduced was omentum and was viable. About six hours after operation, when in excellent general condition, she began to have profuse persistent evacuations of blood, from which she died twelve hours later.

At autopsy all peritoneal surfaces and contents were found normal but the entire mucous surface of the ascending and transverse colon were extremely congested and swollen with small sloughs and ulcers deeply placed. In and below this portion the bowel was full of blood. The imprisoned omentum had drawn upon the colon so that its vessels were occluded by the displacement and tension, and the affected portion of bowel was asphyxiated.

If the case requires operation there are dangers in addition to the foregoing. The intestine may be accidentally injured. The anatomy is so altered that it may be difficult to avoid doing so. In the presence of sloughing, suppuration or fecal extravasation, the peritoneum is necessarily soiled, in which case the patient usually recovers, if proper technique is used, but not always. There must be shock added to that already present. Hemorrhage, the anesthetic and post operative intestinal obstruction are less frequently critical.

When resection has been done, failure of intestinal anastamosis is comparatively common and is almost always fatal. When an artificial anus has been left death has not seldom followed within a few days or weeks from interference with nutrition and from exhaustion, due to constant care of the discharges and pain from the excoriation of the skin they cause, especially if the bowel opening is in the upper part of the intestinal tract. And after all that, there is to be an operation for its closure, which is more dangerous than any ordinary laparatomy. Of those who recover from strangulated hernia with an artificial anus, very few live to recover from that.

Of those who die from strangulated hernia, the greatest number succumb to sepsis at the time of illness; the next to inaction of the bowels, due to the return into the abdomen of a section of the intestine, not dead but paralysed, in which the mucous coat ulcerates, and through which infection enters the circulation and the peritoneal cavity; the next to the results of fecal fistula, which are exhaustion and secondary operations.

One vital point must never be disregarded. It applies to every case whether reduced by taxis or operation. It is, that reduction is by no means sufficient, and until assured of the return of bowel action, the anxiety is scarcely lessened. He who is satisfied when he has put the mass out of sight, is an ostrich with his head in the sand. Unless he has good reason to believe that bowel function will be restored, he should not rest well until it is.

The causes of death in strangulated hernia are principally as follows: Without operation, shock; with or without operation, general peritonitis by continuity from the infected sac and hernia; general peritonitis, from leakage of feces; general peritonitis from sloughing of intestine returned by taxis or after incision; paresis without sloughing, of portion returned, resulting in ileus, septic peritonitis, general septicemia and sometimes fatal hemorrhages into the bowel; asphyxia, in portions of the intestine at a distance from the hernia with necrotic ulcers, also resulting in systemic infection, ileus, septic peritonitis and fatal hemorrhage; with operation, accidental injury to intestine; accidental soiling of peritoneum from infection present before operation; added shock; hemorrhage; anesthesia; intestinal obstruction from post operative adhesions; failure of anastomosis; artificial anus; operation for closure of artificial anus.

The foregoing statements make plain: That taxis should never be attempted after the first few hours. If the hernial tissues have become weak and fragile, they are easily bursted, and if they have become paralysed their return to the abdomen will be a fatal success. That hernia should be remembered and every patient examined for it, if his symptoms allow a possibility of such a cause, and immediately after failure of gentle taxis, if within eighteen hours of strangulation and without taxis; if later in the attack, he should be operated upon. That fecal fistula, besides its distressful immediate consequence has also a large mortality and during operation measures that avoid it even by considerably increasing present risk, if not too severe, are safer for the patient; and that conservatism in its best sense requires a radical cure of all ordinary hernia as soon as discovered instead of treatment by trusses, some cases in young children excepted.

It seems ridiculous to urge upon twentieth century physicians to diagnose hernia, to avoid injuring the intestine by taxis and to secure relief early, but in my experience a majority of cases referred for operation have been detained for treatment until the bowel was dead, and a large minority have existed unrecognised or neglected for nearly a week, many of them sloughing.

525 LAKE AVENUE.

DISCUSSION.

Dr. S. B. SEARS, Syracuse.—I have been very much interested in Dr. Jones's paper, and I think that it is a great error, in regard to attributing the cause of death in strangulated hernia. We are apt to say that if a man is brought upon the operating table, and he succumbs, that the cause of death is the operation, regardless of the conditions under which we operated. Many come to us when the bowel is gangrenous, and when they are completely exhausted. I saw recently a case, perhaps a year ago, where the patient had been brought into the hospital with a hernia that had existed for only a week. During that time she had no movement of the bowels. The operation was performed, and after forty-eight hours there was no untoward effect. She had a good pulse and temperature, after taking away some eight inches of the intestine, and yet that woman died at the end of the third day. I had an autopsy performed upon the woman and the abdomen was clean, the womb was fresh, there was no sign of infection at the womb, and I have a specimen of the intestine with me which shows an absolutely perfect operation. We had there a perfect union of the intestine and a perfectly normal recovery from the anesthetic apparently, and forty-eight hours after operation her pulse was less than a 100 and her temperature 99°, and yet she sank and died. I believe she would have died just the same whether the intestine was taken out or not.

I operated on a man a few days ago, in the afternoon, toward evening, and found the intestine absolutely black. I do not believe we are justified in attributing all deaths to operations simply because an operation has taken place. Another case was a lady, whom I operated on several years ago, who had a hernia, and who wore a truss. I saw her several days later and found the hernia was not reduced. It was a femoral hernia, and the truss was very tight. I operated and reduced the hernia, and four or five hours afterward the woman became black in the face, and died before I could get to her.

Here is a specimen which I wish to show you. It was some sixty odd hours after operation, and shows the line of union in this intestine, and there was absolutely no infection following it that you could find. I am heartily in sympathy with Dr. Jones's paper, and I believe if we would operate earlier and without so much manipulation we would have a far greater percentage of recoveries.

Dr. A. L. BEAHAN, Canandaigua.—The paper is an excellent one, and very comprehensive of the subject. The postponement of cases that cannot be reduced brings about a great amount of danger, and when they are reduced the danger is still great because of the probability that it is reduced in such a way that strangulation will result. It seems to me that an operation for strangulated hernia should be much more common, and one of the reasons is that there is a sepsis beginning very early. Take an old man who dies from an operation for strangulated hernia; it ought not to be attributed to the operation when we know that there is septic peritonitis developed in the case. If in this case an open operation was done and a proper form of drain used, it seems as if some of those cases might be saved. In a general way the open operation is preferable—the abdominal operation—to the common one of following the inguinal canal. I do not see any more danger in an open operation, and dissecting the bowel, knowing what you are doing. There is no doubt that local application of ice and injection of morphin are only temporarily expedients, and should never be regarded in any other light.

